# Cytomegalovirus-induced embryopathology: mouse submandibular salivary gland epithelial-mesenchymal ontogeny as a model

**DOI:** 10.1186/1471-213X-6-42

**Published:** 2006-09-07

**Authors:** Michael Melnick, Edward S Mocarski, George Abichaker, Jing Huang, Tina Jaskoll

**Affiliations:** 1 Laboratory for Developmental Genetics, University of Southern California, Los Angeles, CA 90089-0641, USA; 2 Department of Microbiology and Immunology, Stanford University School of Medicine, Stanford, CA 94305-5124, USA

## Abstract

**Background:**

Human studies suggest, and mouse models clearly demonstrate, that cytomegalovirus (CMV) is dysmorphic to early organ and tissue development. CMV has a particular tropism for embryonic salivary gland and other head mesenchyme. CMV has evolved to co-opt cell signaling networks so to optimize replication and survival, to the detriment of infected tissues. It has been postulated that mesenchymal infection is the critical step in disrupting organogenesis. If so, organogenesis dependent on epithelial-mesenchymal interactions would be particularly vulnerable. In this study, we chose to model the vulnerability by investigating the cell and molecular pathogenesis of CMV infected mouse embryonic submandibular salivary glands (SMGs).

**Results:**

We infected E15 SMG explants with mouse CMV (mCMV). Active infection for up to 12 days *in vitro *results in a remarkable cell and molecular pathology characterized by atypical ductal epithelial hyperplasia, apparent epitheliomesenchymal transformation, oncocytic-like stromal metaplasia, β-catenin nuclear localization, and upregulation of *Nfkb2*, *Relb*, *Il6*, *Stat3*, and *Cox2*. Rescue with an antiviral nucleoside analogue indicates that mCMV replication is necessary to initiate and maintain SMG dysmorphogenesis.

**Conclusion:**

mCMV infection of embryonic mouse explants results in dysplasia, metaplasia, and, possibly, anaplasia. The molecular pathogenesis appears to center around the activation of canonical and, perhaps more importantly, noncanonical NFκB. Further, COX-2 and IL-6 are important downstream effectors of embryopathology. At the cellular level, there appears to be a consequential interplay between the transformed SMG cells and the surrounding extracellular matrix, resulting in the nuclear translocation of β-catenin. From these studies, a tentative framework has emerged within which additional studies may be planned and performed.

## Background

Nearly 75 years ago, Farber and Wolbach [1] reported that postmortem examination of infants less than 1 year of age often revealed large cells containing intranuclear and cytoplasmic inclusion bodies in submandibular salivary glands and, less frequently, in livers, lungs, kidneys, pancreas, and thyroid. The large cells ("cytomegalia") were found in acini and ducts of the affected submandibular salivary glands, and the ducts were often dilated. It was noted that the inclusions were similar to those found in diseases due to "filtrable viruses." Twenty-five years later, human cytomegalovirus (CMV) was isolated [2,3]. By a decade or so after isolation, it was quite apparent that congenital infection with CMV was common and had variant adverse consequences, from asymptomatic viruria to lethality[4].

CMV is an enveloped, double-stranded DNA betaherpesvirus which has been characterized in a large number of mammalian species including humans and mice [5]. The virus has a slow replication cycle, is species specific, and demonstrates particular tropism for salivary glands, and, to a lesser extent, other tissues (lung, kidney, liver, spleen, bone marrow, heart, brain, placenta) [6,7]. In infected newborns, CMV establishes a long-lasting persistence in salivary glands and the virus is shed in saliva for months to years before termination of productive infection and establishment of latency [8].

It is estimated that about 2% of liveborn infants are congenitally infected. About 10–20% of this group have newborn symptoms, and most of these infants will exhibit subsequent abnormalities of the central nervous system (CNS): microcephaly, mental retardation, deafness, and blindness [9-11]. These estimates represent the prevalence of infection and phenotypic outcomes at birth and beyond, not the incidence of infection and associated outcomes during the whole of gestation, particularly during the highly ontogenic first trimester. Unfortunately, the effect of CMV infection on *early *human embryogenesis is uncertain because human studies of early malformation and CMV infection are small, retrospective and temporally truncated [7,12-16]. Nevertheless, mouse models clearly demonstrate that CMV disrupts early organ and tissue development [17-21].

Since mouse CMV (mCMV) has many features in common with human CMV (hCMV) infection, the mouse model has been widely employed to understand the pathogenesis associated with acute, latent, and recurrent infections [20]. When mCMV is introduced into the placenta, the frequency and types of birth defects will depend on the gestational age of infection. Baskar et al. [17-19] have consistently observed substantial fetal loss (reduced litter size and resorbed embryos), fetal growth retardation, and fetal dysmorphogenesis, particularly of the craniofacial complex. Using *in situ *hybridization and immunohistochemistry, they observed that viral sequences and antigens were primarily localized to the brain and salivary glands of malformed craniofacies.

Subsequently, Tsutsui [21] reported that viral antigen-positive cells were abundant in the mesenchyme of the oral and nasal cavities, and in the mesenchyme around the brain. He postulated that mesenchymal infection is the critical step in disrupting organogenesis. If so, organogenesis which is highly dependent on epithelial-mesenchymal interactions (salivary gland, lung, kidney, pancreas, brain, etc.) would be particularly vulnerable to early mCMV infection, and this may explain the frequent fetal demise. In the present study, we chose to model this vulnerability by investigating the cell and molecular pathogenesis of mCMV infected mouse *embryonic *submandibular salivary glands (SMGs).

Mouse SMG development is initiated with a thickening of the oral epithelium of the mandibular arch around embryonic day 11.5 (E11.5) and is best conceptualized in stages as branching morphogenesis forms the ductal system and presumptive acini [22,23]. SMGs being a primary target organ for mCMV replication, with little known about the susceptibility of embryonic tissues, we infected *Canalicular *(E15) SMG explants with salivary gland-derived mCMV. Active infection for up to 12 days *in vitro *results in a remarkable cellular and molecular pathology characterized by atypical ductal epithelial hyperplasia, apparent epitheliomesenchymal transformation, oncocytic-like stromal cell metaplasia, β-catenin nuclear localization, and upregulation of *Nfkb2*, *Relb*, *Il6*, *Stat3*, and *Cox2*. Rescue with an antiviral nucleoside analogue indicates that mCMV replication is necessary to both initiate and maintain SMG pathogenesis.

## Results

Embryonic submandibular salivary glands (SMGs) at E15, exposed to mCMV for up to 12 days *in vitro*, exhibit a singular pathologic phenotype, with dramatic cellular (Figs. 1, 2, 3, 4, 5, 6, 7, 11) and transcriptional (Table 1) changes.

**Figure 1:**
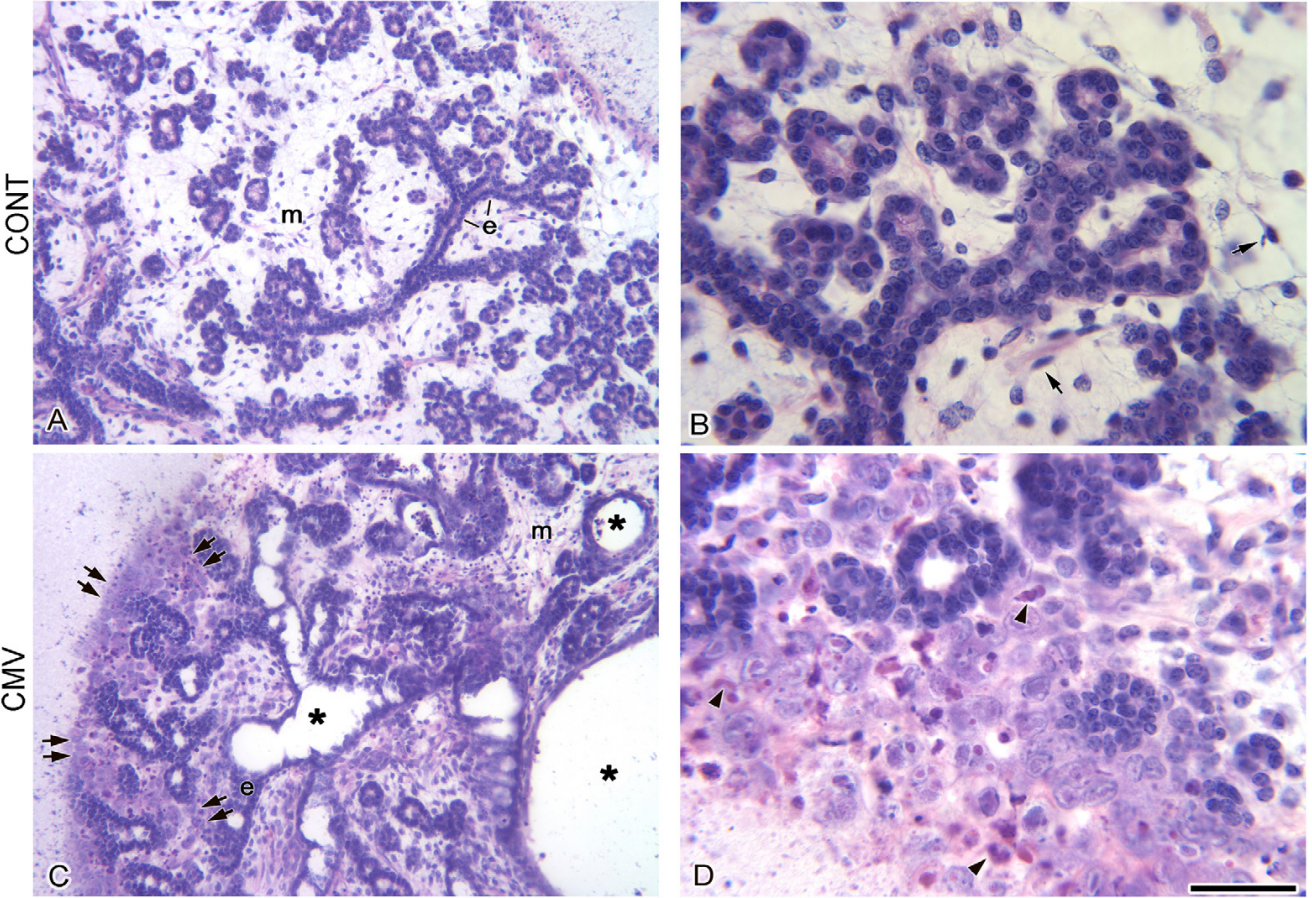
Histopathology of mCMV-infected E15 + 6 SMGs. A, B. E15 + 6 SMGs have achieved the *Terminal Bud *Stage, consisting of ductal and terminal bud epithelia (e) which surround distinct lumina. The epithelial component is embedded in loosely-packed mesenchyme (m) sparsely populated by fibroblasts (arrows). C, D. E15 + 6 SMGs infected with 100,000 PFU mCMV exhibit a marked decrease in branching epithelia and greatly dilated lumina (*); clusters of large, basophilic, pleiomorphic cells, often with inclusion bodies (arrowheads), are seen in peripherally-localized mesenchyme (double arrows); centrally-localized cells retain their fibroblastic morphology (m). Bar: A, C: 100 μm; B, D: 20 μm.

**Figure 2:**
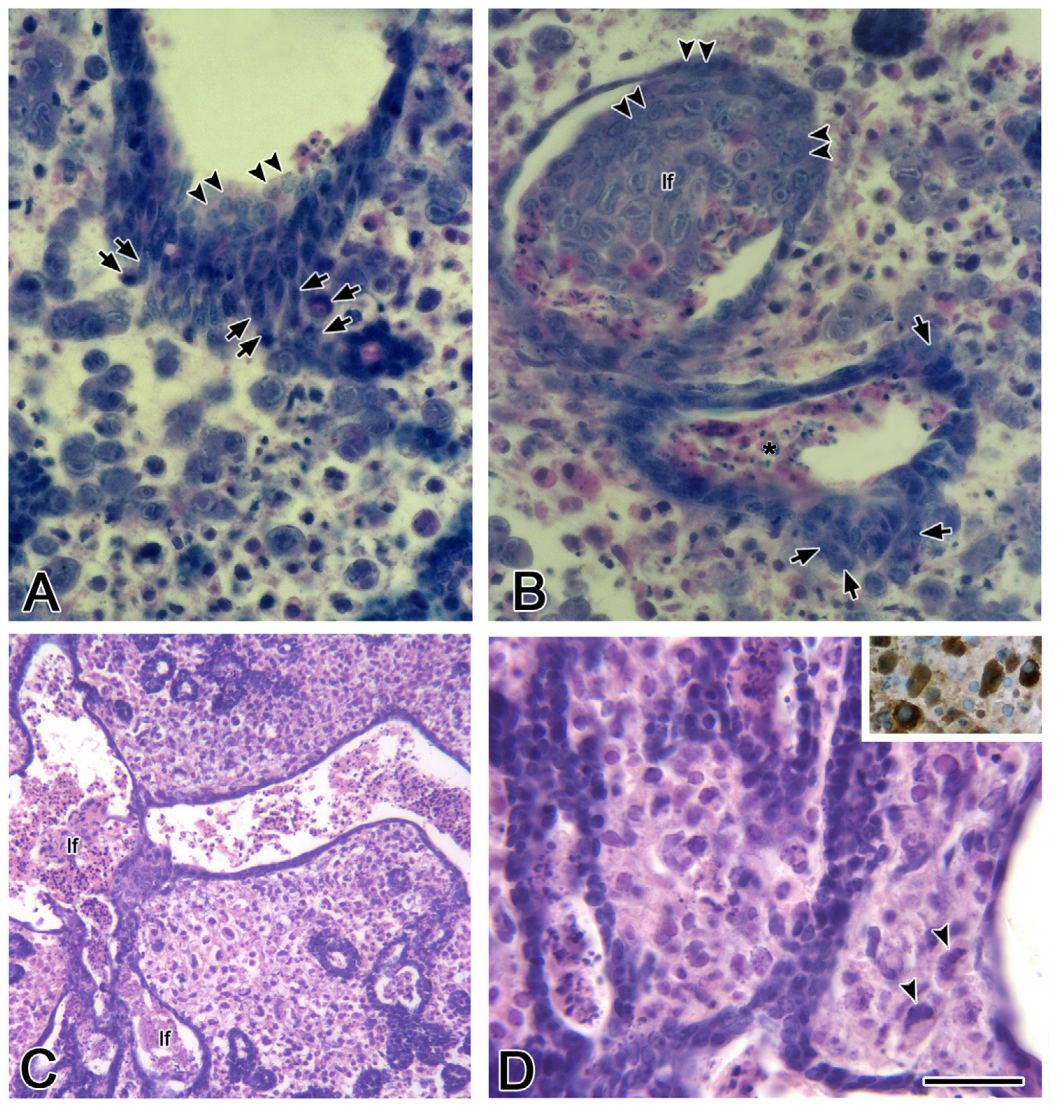
Morphology of progressive mCMV infection in SMGs. A, B. In E15 + 10 mCMV-infected SMGs, the stromal cells are composed of 2 distinct cell types: large basophilic round cells and smaller eosinophilic cells. Basophilic cells can be seen emigrating from the epithelium into the stroma (arrows) and invaginating into the lumina (double arrowheads). Note the presence of pycnotic cells in ductal lumina (*). C-D. E15 + 12 mCMV infected SMGs exhibit a further decrease in branching epithelia and the large, dilated lumina are partially filled with eosinophilic lumina-filling cells (lf). The stroma is entirely composed of large polygonal cells with darkly staining nuclei, eosinophilic cytoplasm, and the frequent presence of inclusion bodies (arrowheads). Insert: Giant polygonal cells exhibit an increase in ATP synthetase protein (brown color), a marker of mitochondrial activity. Bar: A, B, D: 20 μm; C: 100 μm.

**Figure 3:**
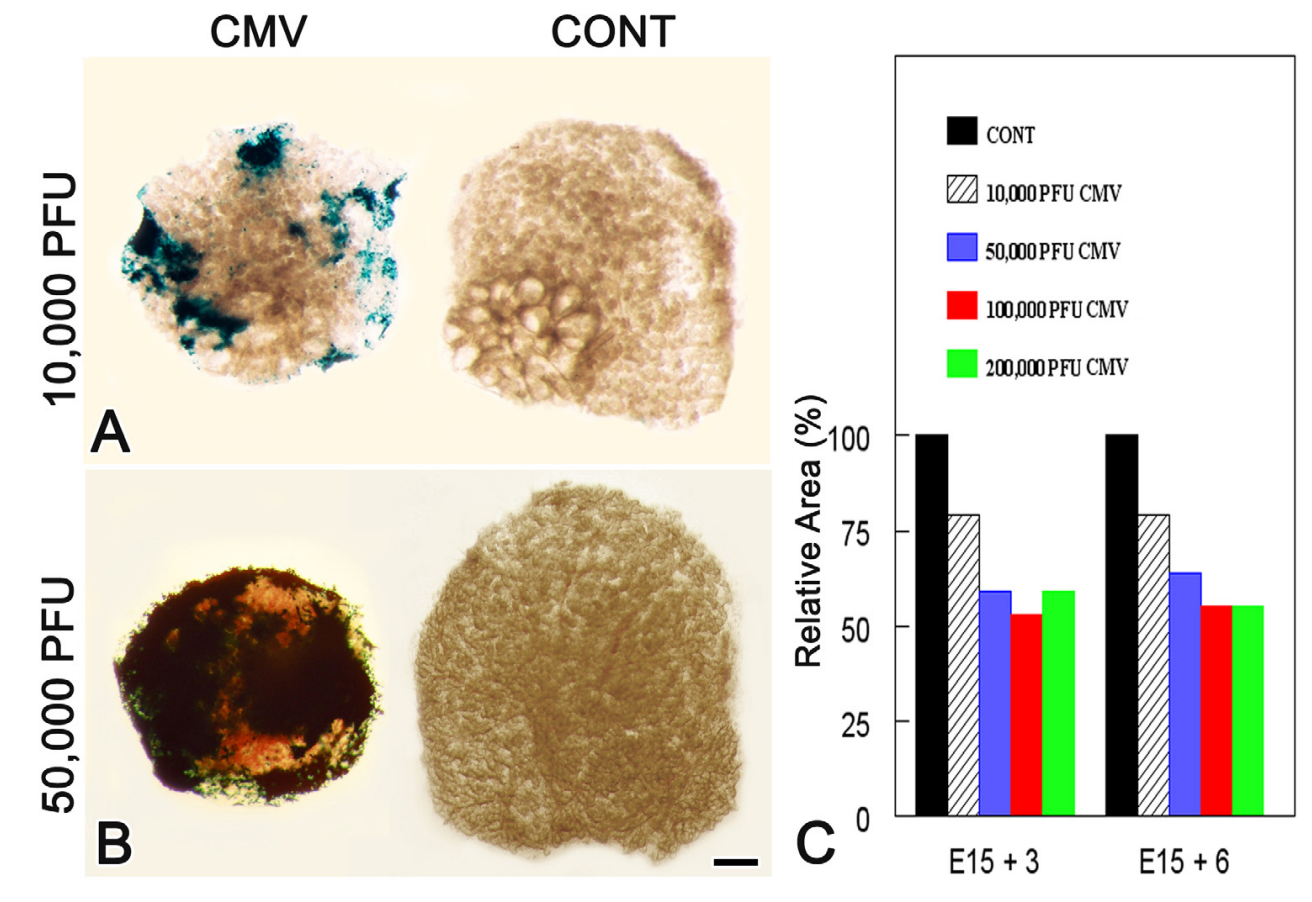
Dose-dependent effect of mCMV-infection on embryonic SMG development. E15+6 SMGs infected with 10,000 PFU mCMV (A) exhibit modest β-gal staining (viral presence) in the periphery, whereas SMGs infected with 50,000 PFU mCMV (B) exhibit staining almost completely throughout the gland. No β-gal staining is seen in control (CONT) SMGs. Bar:50 μm C. Relative to controls, there are significant (P < 0.01) declines in the size of mCMV-infected SMGs (as measured by area); this decline is dose dependent (P < 0.01). Comparisons of control and infected SMGs were based on matched pairs (right v. left in the same embryo). Samples sizes for each bar ranged from 6 to 29 matched pairs. True differences were determined by matched-pairs t-test. See text for details.

**Figure 4:**
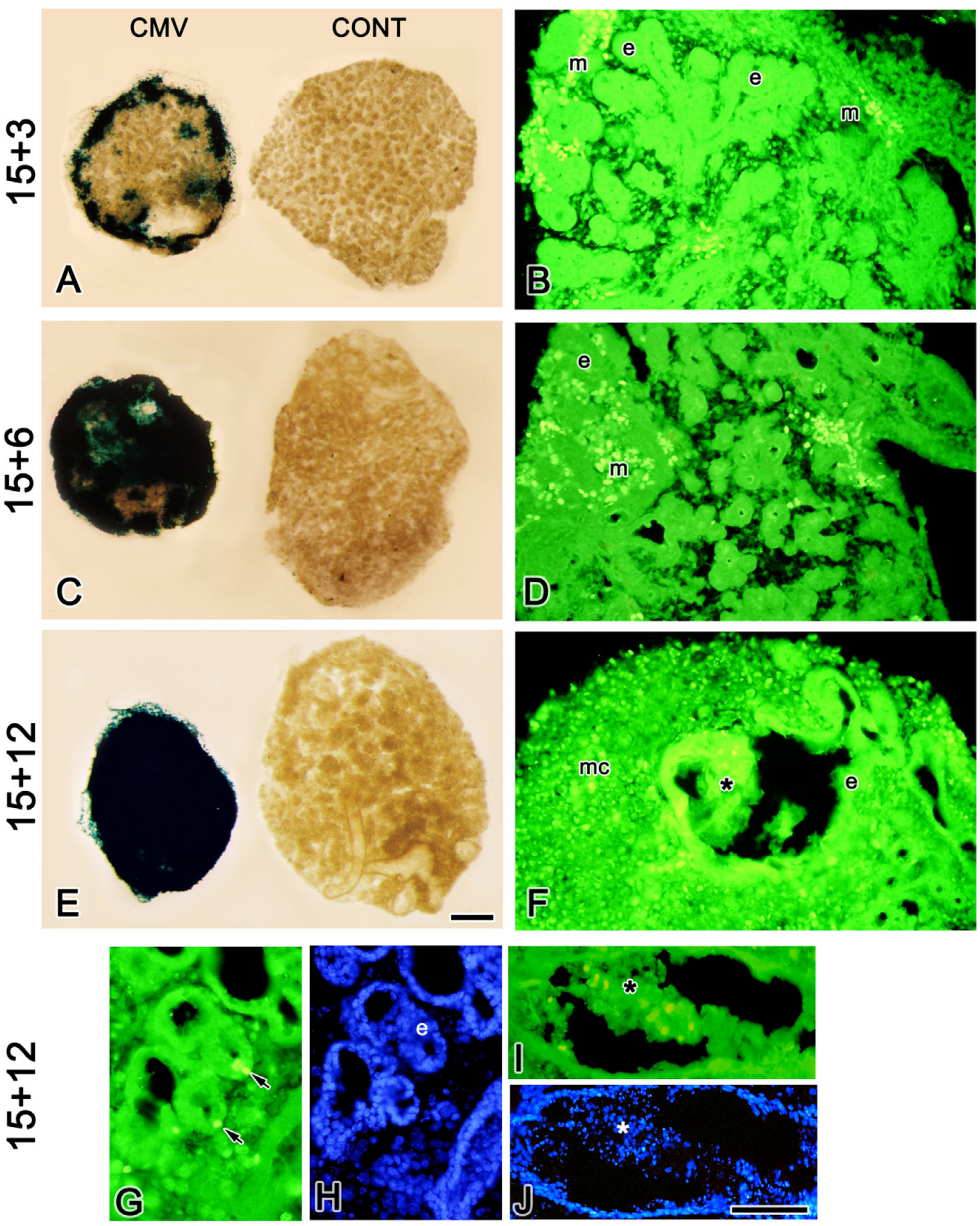
Time-dependent increase in mCMV infection of cultured SMGs. A, C, E. β-gal (mCMV) staining of SMGs demonstrates a marked increase in viral infection with increased days in culture. B, D, F, G, H. Immunolocalization of viral IE1 protein. In E15 + 3 (B) and E15 + 6 (D) mCMV-infected SMGs, IE1 protein is localized to mesenchymal cells (m) and not epithelial cells (e). F, G, I. In E15 + 12 mCMV-infected SMGs, IE1 protein is seen in metaplastic stromal cells (mc), in a few epithelial cells (arrows) and in lumen-filling cells (*). H. J. DAPI staining of E15 + 12 mCMV-infected SMGs shown in G, I. Bar: A: 62 μm; B-F: 50 μm; G, H-J: 25 μm.

**Figure 5:**
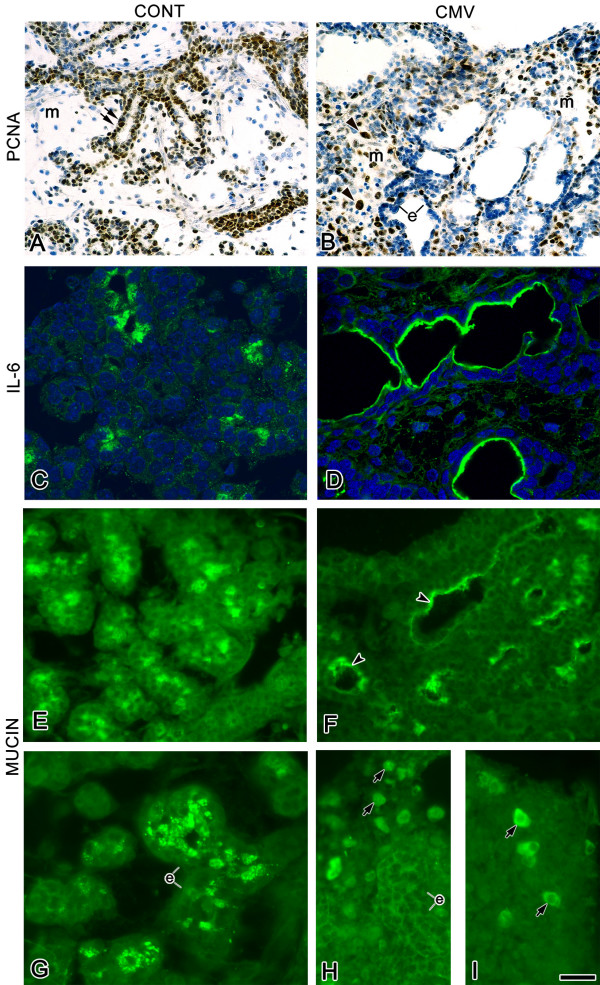
mCMV infection, cell proliferation, IL-6 expression, and mucin expression. A, B. Cell proliferation. Cell proliferation was determined by the distribution of PCNA (brown color). In control E15 + 6 SMGs (A), PCNA positive nuclei are primarily seen in branching epithelia (double arrows) and rarely in mesenchyme (m). With mCMV infection (B), PCNA-positive nuclei are primarily seen in mesenchymal cells (arrowheads) and, to a lesser degree, in epithelial cells (e). C, D. Immunolocalization of IL-6. A substantial increase in immunodetectable IL-6 is seen in mCMV-infected SMGs ductal epithelia (D) compared to controls (C). E-I. Immunolocalization of mucin protein. In E15 + 6 (E) and E15 + 12 (G) SMGs, mucin is localized to the cytoplasm of terminal bud epithelia (e). In mCMV-infected E15 + 6 SMGs (F), there is an increase in mucin localized to epithelial apical surfaces surrounding dilated lumina (arrowheads). By day 12 (H, I), mCMV-infected SMGs are characterized by a notable decline in epithelial-localized mucin; however, mucin is found in a subpopulation of metaplastic stromal cells (arrows). Sections C, D were counterstained with DAPI. Bar: A, B: 23 μm; C-F: 20 μm; G-I: 16 μm.

**Figure 6:**
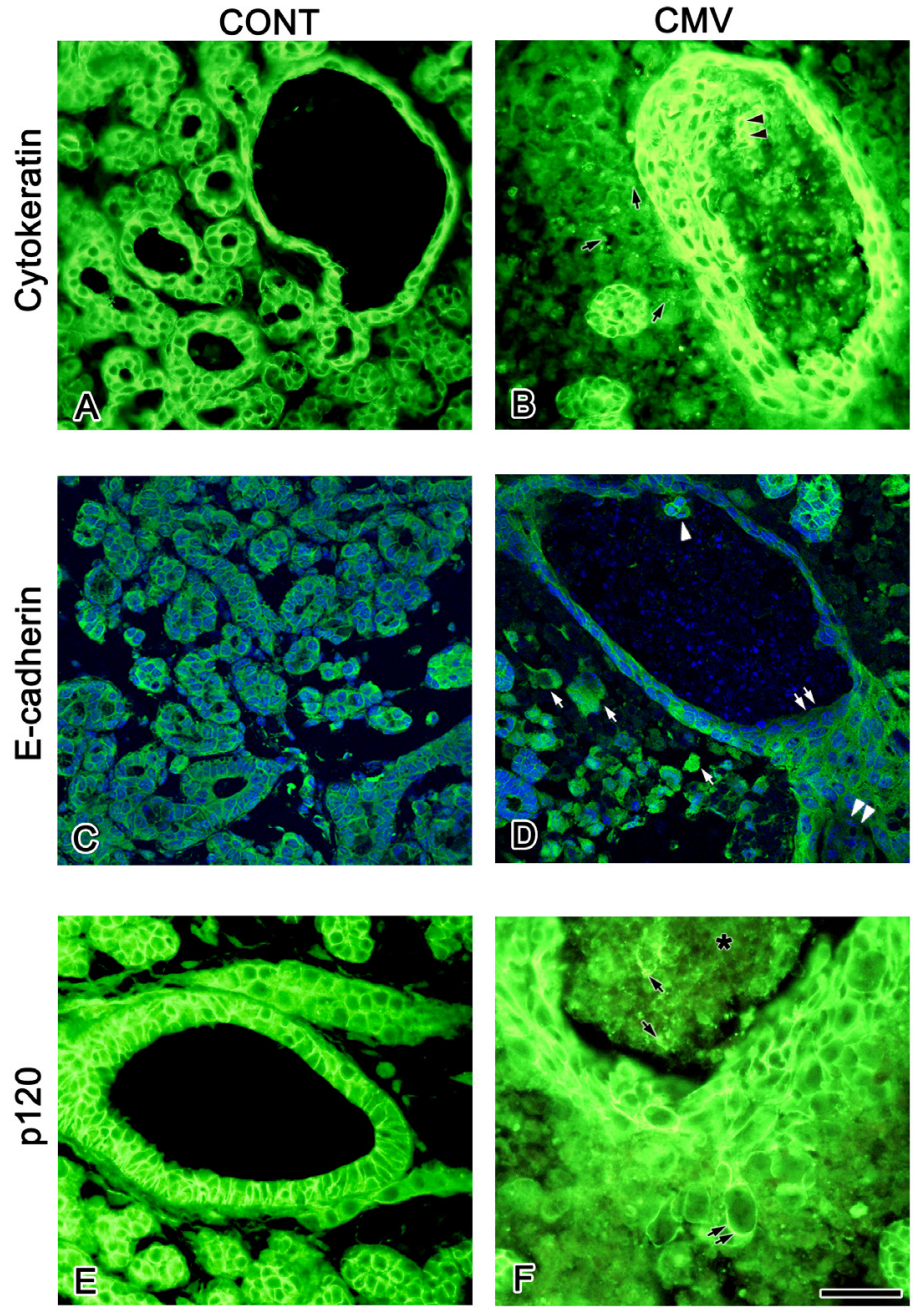
Cell characterization: cytokeratin, E-cadherin, p120. A, B. In control SMGs (A), cytokeratin is immunodetected in branching epithelia and not in mesenchyme; with mCMV infection (B), cytokeratin is detected in the abnormal epithelia, in lumen-filling cells (double black arrowheads), and in the cytoplasm of stromal cells (black arrows) adjacent to pseudostratified epithelia. C, D. In control SMGs (C), E-cadherin is localized solely to epithelial cell membranes. In mCMV infected SMGs (D), a decrease in E-cadherin immunostain is seen in epithelial cells facing the lumina (double white arrows) and in pseudostratified epithelia facing the stroma (double white arrowheads); E-cadherin is also localized to membranes of lumina-filling cells (white arrowhead) and to the cytoplasm of some metaplastic stromal cells (white arrow) adjacent to abnormal epithelia. E, F. p120 localization. In control SMGs (E), p120 is detected adjacent to epithelial plasma membranes and is absent from mesenchyme. With mCMV infection (F), p120 is seen in all epithelia, as well as in cells emigrating from ductal epithelia to stroma (double black arrow) and lumen-filling cells (black arrows). C, D were counterstained with DAPI. * lumen-filling cells. Bar: A, B, E, F: 20 μm; C, D: 27 μm.

**Figure 7:**
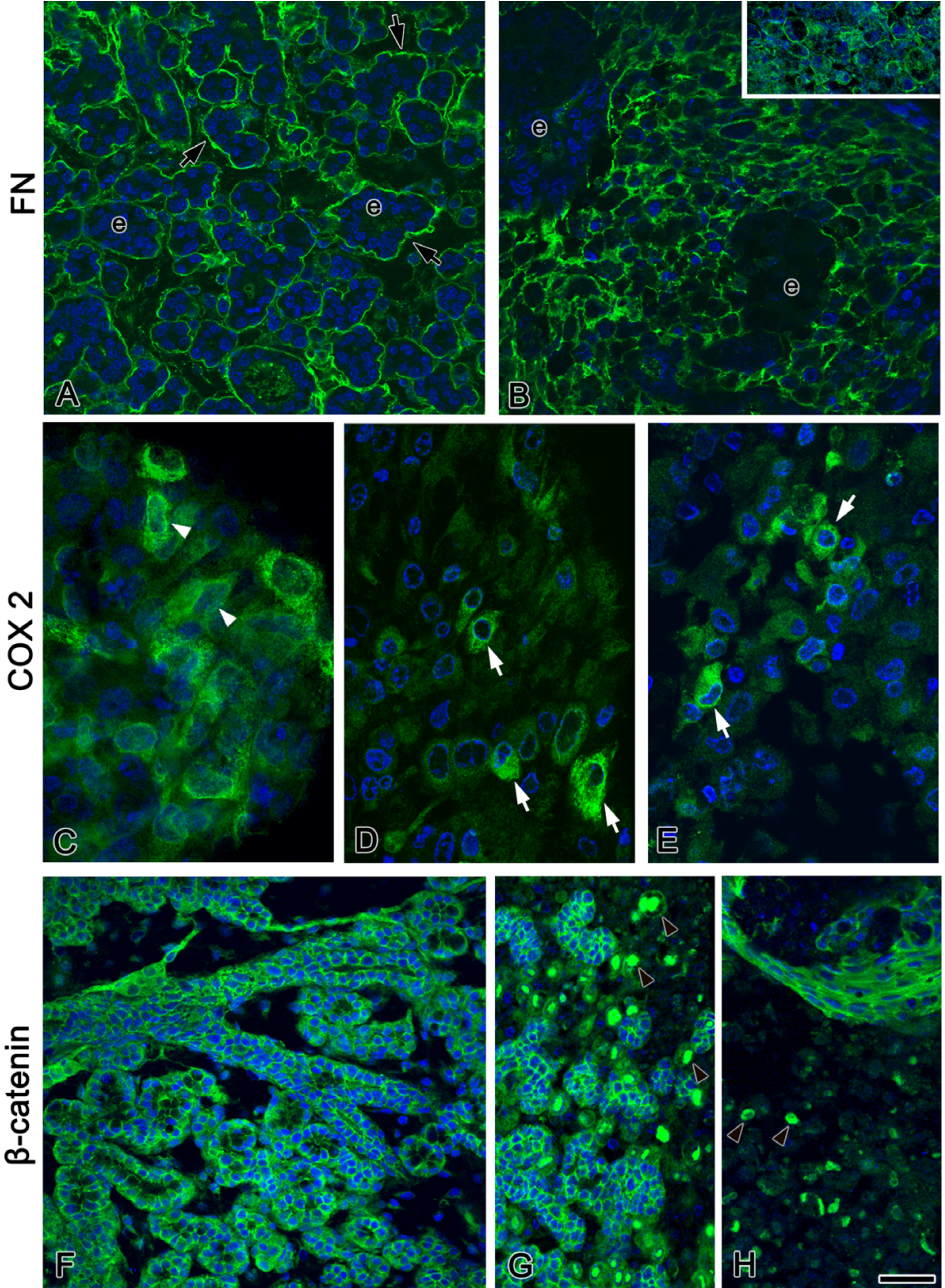
Cell characterization: fibronectin, α5β1 integrin, COX-2, and β-catenin. A, B. In control E15 + 12 SMGs (A), fibronectin (FN) is primarily localized to epithelial (e) basement membranes (arrows). In mCMV-infected SMGs (B), FN surrounds individual stromal cells and there is a marked decrease of FN in epithelial basement membranes. α5 integrin (B, insert) and β1 integrin (data not shown) are similarly localized. C-E. COX-2 is localized in E15 + 3 (C) mCMV-infected SMG fibroblasts (white arrowheads) and in metaplastic stromal cells (white arrows) of E15 + 6 (D) and E 15 + 12 (E) mCMV-infected SMGs. F-H. In control E15 + 12 SMGs (F), β-catenin is immunodetected in the cytoplasm adjacent to epithelial, but not mesenchymal, plasma membranes. With mCMV infection (G, H), β-catenin is seen in intact epithelia, as well as in nuclei of metaplastic stromal cells (arrowheads). All sections were counterstained with DAPI. Bar: 20 μm.

**Table 1:** mCMV Modulation of Embryonic SMG Gene Expression

**Gene**	**R**	**η**
Tgf/β1	0.84	0.11
Tgf/β2	0.67	0.40
Tgf/β3	0.76	0.58
Egfr	1.23	0.43
Tnf	2.10	0.55
Nfκb1	1.15	0.26
**Nfκb2**	1.83**	**0.18**
Rela	1.34	0.46
**Relb**	2.84***	**0.09**
**Il6**	23.02***	**0.62**
**Stat3**	1.72*	**0.29**
Jnk1	0.92	0.55
**Erk1**	0.64*	**0.17**
cmyc	1.78	0.68
Cycd1	0.79	0.71
Cycd2	1.29	0.44
p53	1.00	0.24
Mdm2	1.49	0.78
Casp3	1.39	0.37
Bcl2	0.67	0.67
Ecad	1.10	0.27
βcat	0.80	0.26
Lef1	0.72	0.61
Fn	1.58	0.37
Intβ1	1.42	0.64
Intα5	2.80	0.76
**Cox2**	15.27**	**0.66**

R = mean relative expression ratio= mCMV/control (R is the mean of 3–5 independent experiments)

η = gene expression noise = s_R_/R (where s_R _= standard deviation of R)

* P < 0.05; ** P < 0.01; *** P < 0.001

### Histopathology

After 6 days in culture, E15 SMGs typically progress to the *Terminal Bud Stage*: bilaminar, stratified epithelial ducts and single-layered epithelial terminal buds display distinct lumina, and are embedded in a loosely-packed stroma sparsely populated with fibroblasts (Fig. 1A, B). E15 + 6 SMGs infected with 100,000 PFU mCMV show a marked decline in branching epithelia; duct epithelia is pseudostratified and poorly organized; duct, and perhaps bud, lumina are greatly dilated (Fig. 1C, D). There is a several-fold increase in cellularity of the stroma, particularly at the periphery of the SMG (Fig. 1C). This zone of atypia contains clusters of large, basophilic, pleiomorphic cells, with high nuclear-to-cytoplasmic ratios, prominent nuclei and nucleoli, and frequently inclusion bodies pathognomonic of mCMV infection (Fig. 1D).

As the infection progresses, the stromal cells are composed of two distinct cell types: large basophilic round cells and smaller eosinophilic cells. In cross-section, the basophilic cells appear to be emigrating from the epithelia (Fig. 2A, B) and invaginating into ductal lumina (Fig. 2A, B). By 12 days in culture, there is a further decline in branching epithelia, and the further dilated lumen of the pseudostratified ducts are filled with eosinophilic cells, some living, some apoptotic (Fig. 2C, D). Throughout the gland the stroma is hypercellular and no longer resembles mesenchyme (Fig. 2C, D). The stromal cell type at E15 + 12 is unusual: large polygonal cells with large, darkly staining, nuclei containing prominent nucleoli, densely eosinophilic cytoplasm, and the frequent presence of inclusion bodies (Fig. 2D). This plump, eosinophilic cell type with mitochondrial hyperplasia (Fig. 2D, insert) is suggestive of oncocytic metaplasia [24,25]. Other than in some intraductal epithelial cells, there is scant evidence of cell death in epithelial or stromal cells. This is consistent with the well-documented CMV suppression of cell death [26-33].

### Dose response and time course

The severity of the morphologic changes in SMG development is mCMV dose-dependent (Fig. 3). At 10,000 PFU, SMGs are only moderately infected at the organ periphery, and there is an approximate 25% decrease in gland size (P < 0.01). At 50,000 PFU, SMGs are almost totally infected at the periphery, and about a 40% decrease in gland size (P < 0.001); this differs significantly from the smaller dose (P < 0.01). There are no significant differences between 50,000, 100,000, and 200,000 PFU with respect to SMG size. This is consistent with the fact that viral titers in SMGs exposed to any of these three doses, are not significantly different at E15 + 6 (F_2,9 _= 1.48; P > 0.25).

At 100,000 PFU, the negative effect on branching morphogenesis is evident early. There is a significant 50% reduction in SMG size (P < 0.001) at E15 + 3, and this reduction significantly increases (P < 0.02) through E15 + 12 (Fig. 4A, C, E), as epithelial structures are increasingly replaced by metaplastic stromal cells (Fig 4B, D, F), and viral titers significantly increase with increasing time of exposure (P < 0.05). Immunodetection of mCMV immediate early protein 1 (IE1) reveals that through the first 6 days of culture, mCMV infection is localized to mesenchymal cells (fibroblasts)(Fig 4B, D); by 12 days, the infection is confined mostly to metaplastic stromal cells, but may also be found in a few ductal epithelial cells and in lumen-filling cells (Fig. 4F–J). These data are supported by β-galactosidase localization, an indicator gene product expressed from the virus we used (data not shown). It is important to note that at least through 6 days of culture, the infected fibroblasts are quite distant from much of the affected branching epithelia (Figs. 1C, 4B, 4D), suggesting paracrine factor diffusion from virus-infected areas. In this regard, it is also important to note that, although there is a progressive spread of infection over the 12-day time course, the majority of affected stromal cells remain uninfected throughout the observation period.

### Cell proliferation

The clear decline in branching morphogenesis and increase in stromal cellularity is directly correlated with cell proliferation (Fig. 5). At E15 + 6, *Terminal Bud *Stage SMGs typically exhibit evidence of proliferation mostly in the branching epithelia, and very little in the mesenchyme (Fig. 5A). In mCMV infected SMGs, this pattern is reversed (Fig. 5B); mostly mesenchymal, not epithelial, cells are proliferating.

IL-6 signaling plays an important dual role in SMG development: branching morphogenesis through the SHP-2/Ras mitogenic pathway and ductal maturation through the STAT3 pathway [34]. In uninfected SMGs, IL-6 is expressed in proliferating epithelial cells surrounding the forming lumina (Fig. 5A, C), consistent with its mitogenic pathway. In mCMV infected SMGs, however, IL-6 is more intensely expressed in the **non**-proliferating epithelial cells surrounding dilated lumina (Fig. 5B, D). This finding suggests an upregulation of IL-6 expression and a premature shift to the STAT3 pathway for epithelial maturation. This proposition is supported by our gene expression studies below (Table 1) and by premature SMG terminal differentiation (Fig. 5E, F), as determined by the expression of mucin (Muc10), a SMG-specific marker of epithelial histodifferentiation [35,36]. To wit, in mCMV-infected E15 + 6 SMGs, we see an increase in mucin protein translocated from cytoplasm to epithelial apical surfaces surrounding dilated lumina. In this regard, it is important to note that by E15 + 12, many of the metaplastic stromal cells are also expressing mucin (Fig. 5H–I), consistent with an epithelial origin.

### Cell characterization

SMG epithelium is characterized by cytoplasmic cytokeratin and adherens junctions (Fig. 6). In uninfected SMGs, cytokeratin is exclusively seen in branching epithelia (Fig. 6A). In mCMV infected SMGs, cytokeratin is expressed in the abnormal, dilated ductal structures composed of pseudostratified squamous and lumina-filling epithelia, and more diffusely in the metaplastic stromal cells adjacent to this epithelia (Fig. 6B).

E-cadherin and p120 are important constituents of adherens junctions. In uninfected SMGs, E-cadherin is immunolocalized exclusively to epithelial plasma membranes (Fig. 6C). With mCMV infection, the pseudostratified lumina epithelia display a decline in expressed E-cadherin in cells facing the lumina and in cells facing the stromal space; there is also subsequent cytoplasmic localization of E-cadherin in metaplastic stromal cells adjacent to ductal epithelia (Fig. 6D). In uninfected SMGs, p120 is expressed adjacent to plasma membranes in epithelia (Fig. 6E); in mCMV infected SMGs, p120 is expressed in all epithelia, but markedly less in those cells which appear to be emigrating from ductal epithelia into the stroma (Fig. 6F).

Given these observations (Fig. 6B, D, F), as well as the recent study by Davis and Reynolds [37] of embryonic SMG epithelial dysplasia and E-cadherin deficiency in *p120 *null mice, it is reasonable to suggest that the lumen-filling cells are completely derived, and the metaplastic stormal cells are at least partially derived, from epithelium.

Recent studies indicate unexpected links between fibronectin expression, COX-2 expression, and β-catenin nuclear localization [38,39], as well as between CMV infection and COX-2 expression [40-42]. Thus, we investigated the cellular distribution of fibronectin (FN), α5β1 integrin (FN receptor), COX-2 and β-catenin in *Terminal Bud *Stage SMGs, with and without mCMV infection (Fig. 7). Normally, FN is primarily immunolocalized in ductal and terminal bud epithelia basement membranes (Fig. 7A). With mCMV infection, there is a remarkable shift in FN distribution: FN surrounds individual metaplastic stromal cells, and there is a notable decline in basement membranes (Fig. 7B). A similar change in α5β1 integrin is seen with mCMV infection (Fig. 7B, insert). COX-2 is not expressed in uninfected stromal cells (not shown), but is localized in the cytoplasm of infected fibroblasts (Fig. 7C) and metaplastic stromal cells (Fig. 7D, E) in mCMV infected SMGs. This dramatic change is associated with a change in *Cox2 *transcript level (Table 1). Concurrently, β-catenin exhibits a most significant shift in location with mCMV infection of SMGs. In *Terminal Bud *Stage SMGs, β-catenin (an adherens junction constituent) is immunolocalized to the cytoplasm adjacent to epithelial plasma membranes (Fig. 7F). With mCMV infection, β-catenin is still expressed in intact epithelia, but, more importantly, there is *de novo *expression in the nuclei of metaplastic stromal cells adjacent to that epithelium (Fig. 7G, H). Nuclear localization of β-catenin is indicative of its alternate function as a transcription factor.

### Transcription changes

Functional studies in our laboratory and elsewhere have demonstrated that embryonic SMG organogenesis is regulated through interconnected growth factor, cytokine, and transcription factor-mediated signaling pathways, including EGF, TGF-β, IGF, IL-6, Shh, and FGFs [22,23,34,43-49]. The **hub **of this complex network of parallel and broadly-related pathways is NFκB [44]. In the present study, we employed real-time quantitative PCR to determine transcription changes with mCMV infection (Table 1). The 27 genes chosen include sentinel genes from the signaling network [44], as well as those that characterize cellular changes with mCMV infection (Figs. 5, 6, 7).

The results of these measurements are presented in Table 1. The relative expression ratio (R) is the mean increase or decrease in gene expression in mCMV infected glands compared to uninfected glands. The variation of R is calculated as gene expression noise (η); η is statistically equivalent to the coefficient of variation and ranges from 0 to 1 [50]. The value of η reflects fluctuations in the level of promoter-binding or the abundance of a particular transcription factor, variation in post-transcriptional modifications, and a host of other stochastic events that are characterized as intrinsic or extrinsic noise [50]. In the absence of prohibitively large sample sizes, as η approaches 1 it becomes extremely difficult to detect small, but important, true differences in gene expression levels, should they exist. Of the 27 genes measured, 6 exhibited statistically significant changes in expression with mCMV infection: *Nfkb2, Relb, Il6, Stat3, Erk1, Cox2*. These results are entirely consistent with previous studies [41,44,51,52] and with our immunohistochemical studies (Figs. 5, 6, 7).

### mCMV replication and pathology

There are two key questions about the relationship of viral replication to the subsequent SMG pathology. First, is mCMV replication necessary to initiate the pathogenesis? Second, is mCMV replication necessary to maintain the pathogenesis? To answer these questions, we utilized acyclovir, an antiherpesviral nucleoside active against mCMV [53].

**Figure 8:**
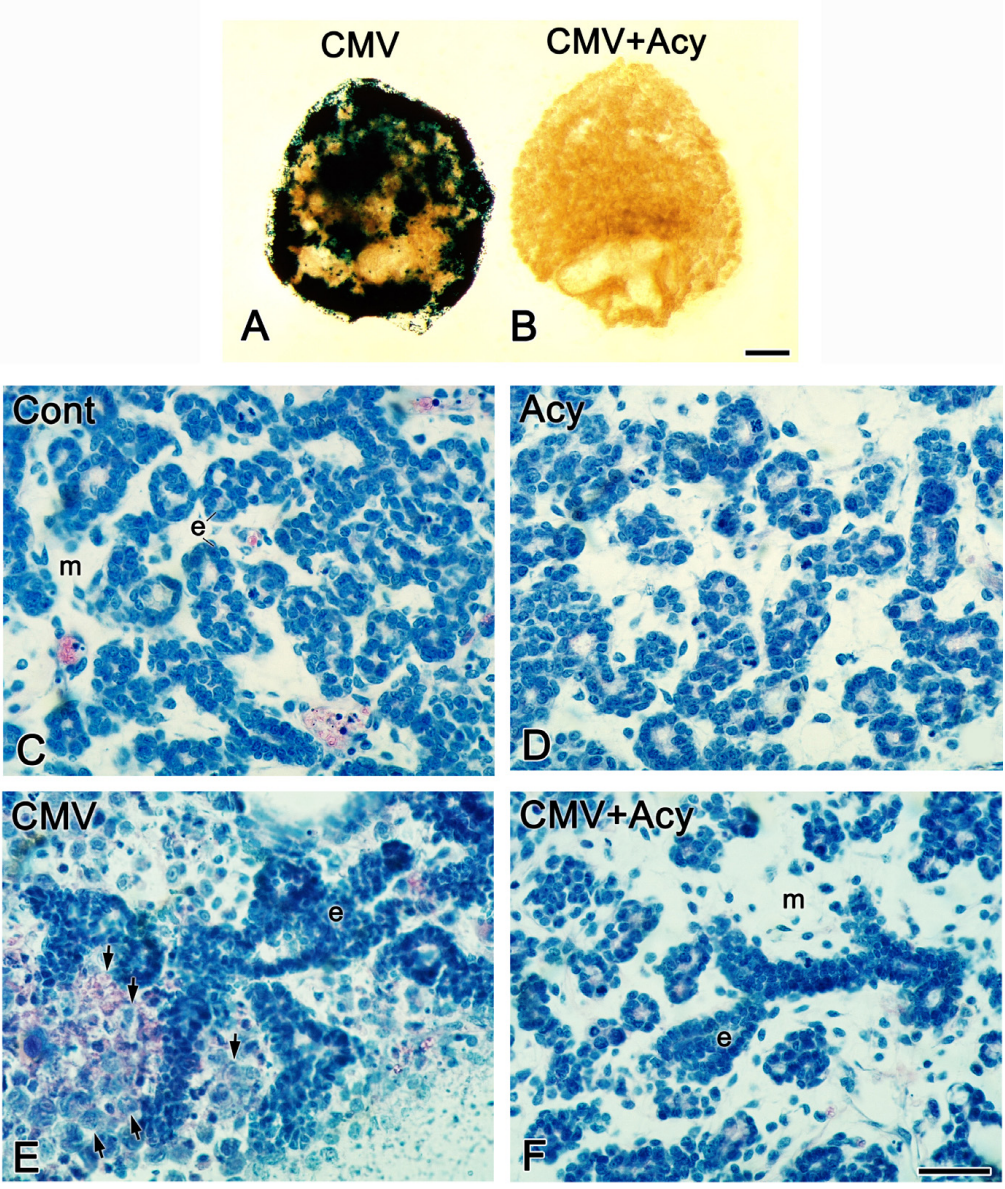
Acyclovir treatment inhibits mCMV replication and rescues the mCMV-induced abnormal phenotype. β-gal (mCMV) staining is seen throughout E15 + 6 SMGs infected with 50,000 PFU mCMV (A) but is absent from SMGs cultured with mCMV + acyclovir (CMV + Acy) (B). C-F. Histological analysis of control (C), acyclovir-treated (Acy) (D), mCMV-infected (E), and mCMV-infected explants treated with acyclovir (CMV + Acy) (F) SMGs. The epithelial (e) and mesenchymal (m) cellular morphology in CMV + Acy glands (F) is similar to that seen in control (C) and acyclovir-treated (D) SMGs. Note that acyclovir treatment of mCMV-infected SMGs (F) maintained the fibroblastic appearance of the mesenchyme (m); typically, mCMV-infected glands (E) exhibit clusters of large, basophilic abnormal cells (arrows) in the periphery. Bar: A, B: 50 μm; C-F: 20 μm.

In the first experiment, E15 SMG explants were infected for 24 hrs with mCMV and then cultured in the presence or absence of acyclovir for an additional 5 days (Fig. 8). Acyclovir treatment, as expected, surpresses mCMV replication (Fig. 8A, B); also, acyclovir treatment results in histologically normal SMGs (Fig. 8C–F) with normal patterns of cell proliferation (data not shown). This outcome is associated with a highly significant mean reduction in SMG mCMV titer from 2.1 × 10^4 ^PFU to 20 PFU (P < 0.01). The replication cycle is not completed by 24 hrs of infection when antiviral treatment was initiated, so it appears that completion of the viral replication cycle beyond DNA replication is critical to the initiation of SMG pathogenesis.

**Figure 9:**
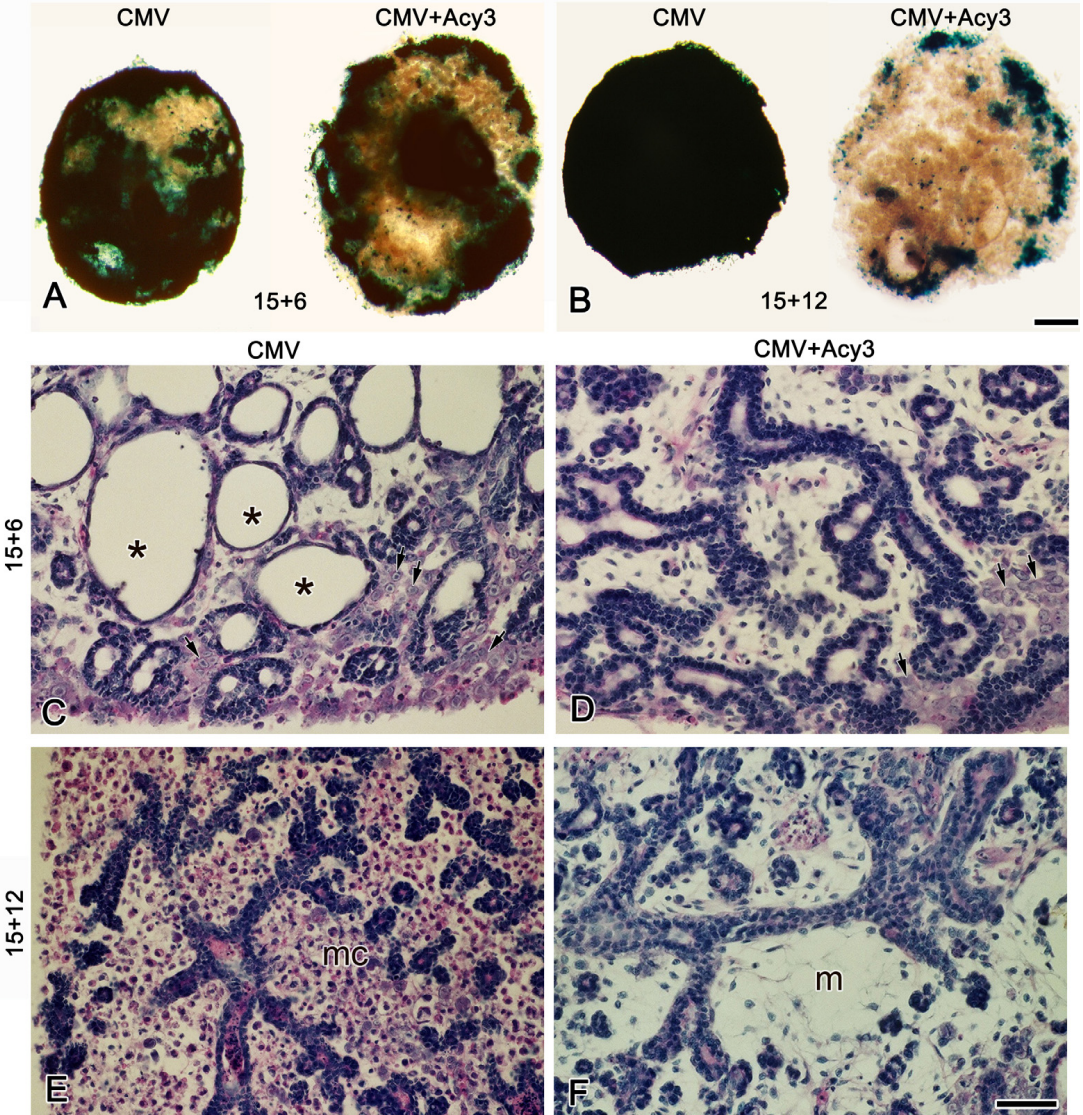
Acyclovir rescues E15 SMGs infected for 3 days with mCMV and cultured for an additional 3 days (E15 + 6) (A, C, D) or 9 days (E15 + 12) (B, C, F) in the presence (CMV + Acy3) or absence (CMV) of acyclovir treatment. On day 6 (A), there is a decrease in β-gal (mCMV) staining in CMV + Acy3 SMGs compared to mCMV SMGs; by day 12 (B), acyclovir treatment results in sparse β-gal staining. At E15+6, with mCMV infection alone (C), abnormal epithelia surround dilated lumina (*) and clusters of atypical basophilic cells are seen in the periphery (arrows); in contrast, acyclovir treatment (D) partially restores the epithelial phenotype to that seen in control SMGs, with fewer atypical mesenchymal cells (arrows). At E15 + 12, acyclovir treatment (F) results in the near normal appearance of epithelia and mesenchyme (m); this markedly differs from the histopathology seen in E15 + 12 CMV-infected SMGs (E). mc-metaplastic stromal cells. Bar: A, B: 50 μm; C-F: 30 μm.

In the second experiment, E15 SMG explants were infected with mCMV for 72 hrs, which allows for complete viral replication, followed by culture for an additional three (E15 + 6) or nine (E15 + 12) days in the presence or absence of acyclovir (Fig. 9). At E15 + 6, there is an evident inhibition of mCMV with acyclovir treatment (Fig. 9A); concomitantly, the SMGs are histologically near normal with only mildly dilated ducts, increased branching, and mostly normal stroma (Fig. 9C, D). By E15 + 12, there is only sparse evidence of detectable mCMV in acyclovir-treated SMGs (Fig. 9B), with histologically normal epithelial branching and the relatively rare appearance of metaplastic stromal cells (Fig. 9E, F). As is normally seen (Fig 5A), cell proliferation is almost exclusively seen in the branching epithelia (data not shown). Thus, eventhough SMGs were subjected to unimpeded mCMV replication for 72 hrs, the impact of reduced replication and spread was sufficient to dramatically reduce viral cytopathology and associated developmental changes. Embryonic cellular memory [54] appears insufficient to maintain progressive pathogenesis.

### NFκB and mCMV-induced pathogenesis

**Figure 10:**
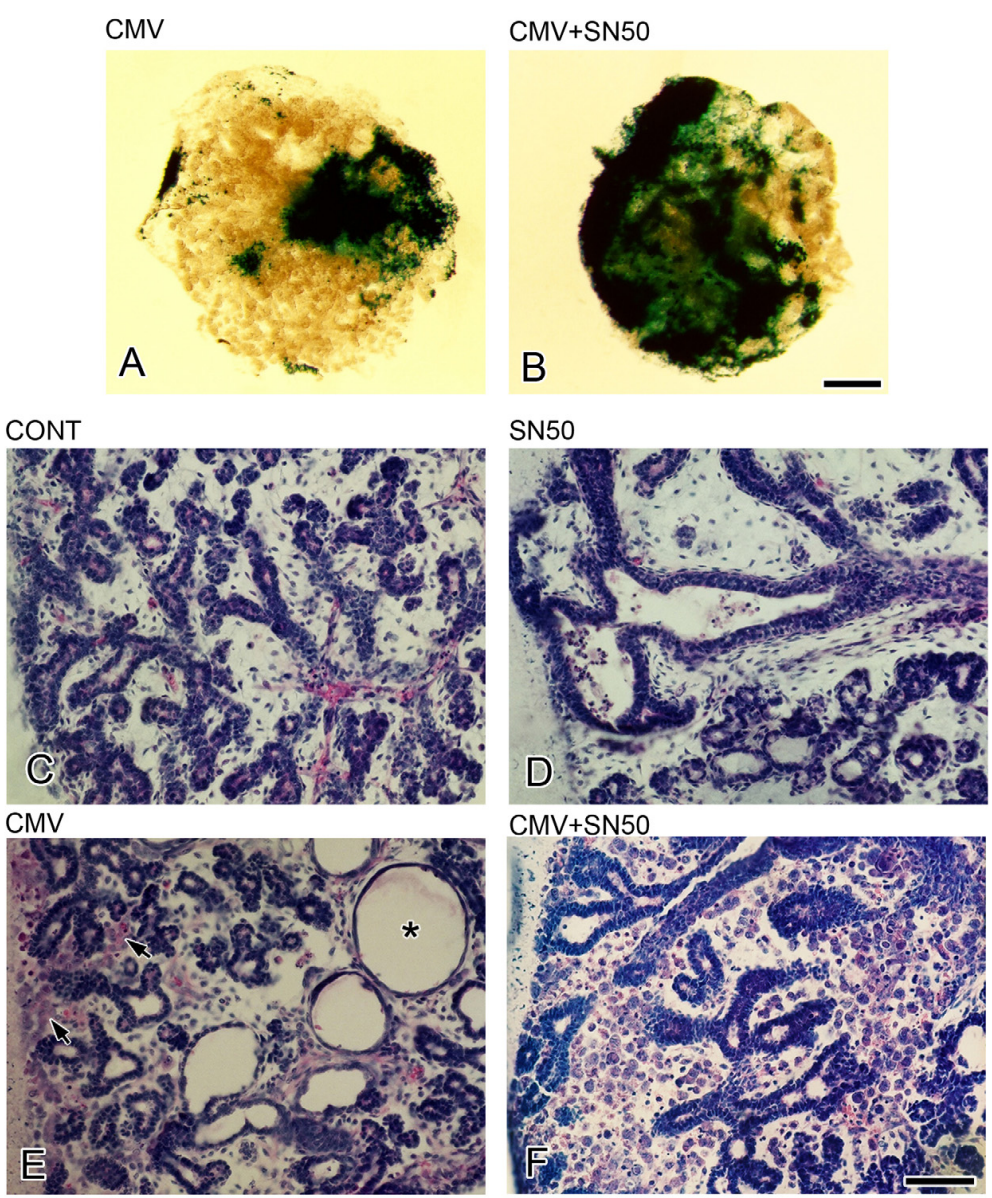
mCMV infection and SN50 inhibition of canonical NFκB nuclear translocation. With mCMV+SN50 treatment of E15 SMGs (B), there is a notable increase in β-gal (mCMV) staining compared to mCMV infection alone (A). Control E15 + 6 SMGs (C) are at the *Terminal Bud *Stage with epithelia surrounding distinct lumina; E15 + 6 SN50-treated SMGs (D) are characterized by a modest decrease in epithelia and larger, somewhat dilated lumina. The glandular morphology of CMV + SN50 (F) differs from that seen with SN50 (D) or mCMV infection (E) alone. mCMV-infected E15 + 6 SMGs (E) exhibit peripherally-localized clusters of large, atypical, basophilic cells (arrows) and greatly dilated lumen (*); by contrast, the stroma of the CMV + SN50 E15 + 6 SMGs is almost entirely composed of polygonal cells with darkly staining nuclei and often eosinophilic cytoplasm, the metaplastic cell type typical of E15 + 12 mCMV-infected SMGs (compare to Fig. 9E). Bar: A, B: 50 μm; C-F: 30 μm.

Evidence indicates that the early CMV protein, IE1, activates canonical NFκB (p50/RelA) by inducing its nuclear localization, rather than transcriptional/translational upregulation [55,56]. Activated NFκB binds to the NFκB recognition sites of viral *ie *genes and a large array of host cell genes. It is reasonable, then, to expect that inhibition of NFκB nuclear localization would moderate mCMV-induced pathogenesis, either through an impact on the virus or host cells. In fact, the results of just such an experiment are quite the reverse of expected (Fig. 10). mCMV infection of E15 SMGs in the presence of SN50, a cell permeable inhibitor of canonical NFκB nuclear translocation, exhibits an early accelerated viral replication (Fig. 10A, B) and SMG dysplasia. At E15 + 6 (Fig. 10E, F), the pathology is more characteristic of the typical mCMV-infected E15 + 12 SMGs (e.g. Fig. 9E).

**Figure 11:**
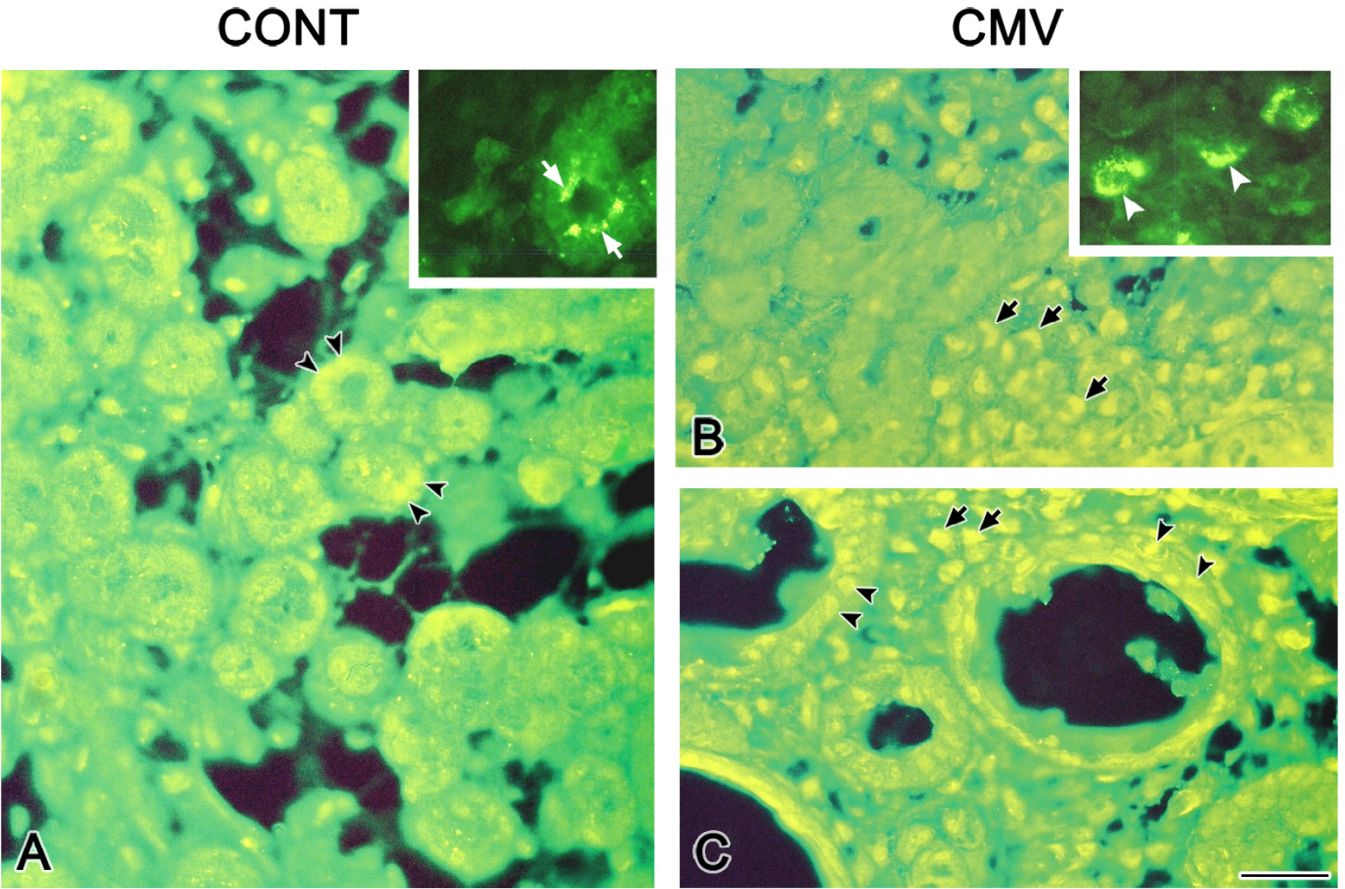
Immunolocalization of RelB and NFκB2 in mCMV-infected E15 + 6 SMGs. A-C. RelB localization. Insert: NFκB2 localization. In control SMGs, RelB (A) (black arrowheads) and NFκB2 (insert, white arrows) are seen in epithelia surrounding forming lumina and not in the stroma. With CMV infection (B, C), RelB is seen in the nuclei of stromal cells (black arrows), many of which exhibit inclusion bodies. RelB is also seen in the nuclei of ductal epithelia (black arrowheads) surrounding enlarged lumina (C). In CMV-infected glands, NFκB2 is seen in the cytoplasm of large stroma cells (insert, white arrowheads) and epithelia surrounding lumina (data not shown). Bar: 50 μm.

These results are consistent with recent studies in human and mouse fibroblast cells grown in culture suggesting that canonical NFκB has paradoxical roles in infected cells [56]. Further, Sonenshein's group [51,52] presents evidence that non-canonical RelB dimers may be more critical to *de novo *host tissue gene expression and pathology. Here we find an upregulation of *Relb *and *Nfkb2 *(Table 1), as well as the nuclear localization of RelB (Fig. 11). Indeed, compensatory upregulation of *Relb *and *Nfkb2 *in the presence of SN50 may ultimately prove to be the explanation for accelerated viral replication and pathology (Fig. 10).

## Discussion

**Figure 12:**
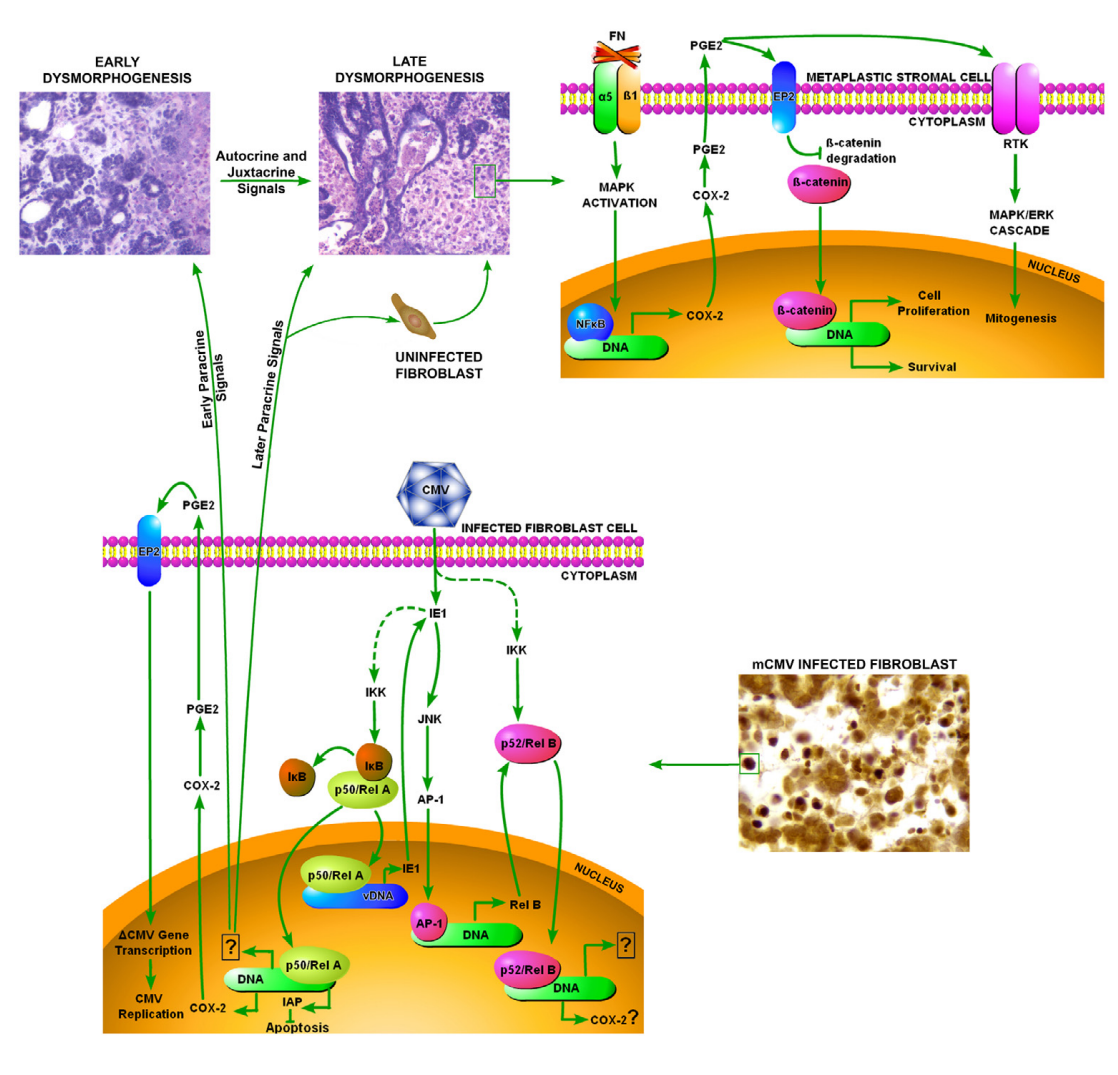
Working model of CMV-induced salivary gland dysmorphology. → Single or multistep stimulatory modification.  single or multistep stimulatory modification of unknown mechanism. ⊣ single or multistep inhibitory modification. RKT: Receptor Tyrosine Kinase. DNA: host DNA. vDNA: viral DNA.

Communication, reciprocal or otherwise, between epithelium and mesenchyme is a critical ontogenic event for many organs and tissues; salivary gland development is a classic example [57]. Since human studies suggest [14,15], and mouse models clearly demonstrate [17,21], that CMV is dysmorphogenic to early organ and tissue development, and since CMV has a particular affinity for embryonic and adult salivary glands [17,58], we investigated the cell and transcriptional affects of mCMV infection on developing mouse embryonic submandibular salivary glands (SMG). Our experimental results provide a portal to the complexity of the pathogenesis. Figure 12 summarizes and *tentatively *models our complex findings in the context of the extant literature, and is explicated below.

With rare exception, viral protein (IE1) is only immunolocalized to stromal cells. After 12 days in culture, mCMV infected E15 SMGs are highly dysmorphic. The epithelial component, normally the principle cell type, is characterized by diminished proliferation, cessation of branching, and a dramatic near absence of terminal buds (future acini). Unlike normal ducts which have a bilaminar structure of stratified basal and luminal cell layers, affected ducts display a disordered pseudostratified epithelium. The lumina are greatly enlarged and filled, to varying degrees, with living and dead cells that appear to have budded off the epithelium alone or in clusters, a phenotype reminiscent of secretory gland neoplasia. These abnormal ductal structures are embedded in a hypercellular stroma that no longer resembles mesenchyme. Indeed, the ductal basement membrane is greatly diminished and epithelial cell migration appears to contribute to the stromal cell population as well. The plump, polygonal-shaped, stromal cells are generally characterized by large, darkly staining nuclei, densely eosinophilic cytoplasm, and mitochondrial hyperplasia. These cells very much mimic oncocytotic metaplasia [24,25].

The proliferating stromal cells in infected SMGs are apparently not homogeneous. One group appears to be epithelial in origin and is characterized early on by cytoplasmic localization of the epithelial proteins cytokeratin, E-cadherin, and p120, and later by epithelial-specific mucin protein and nuclear localization of β-catenin; another group has none of these characteristics and is probably of fibroblast origin. Heterogeneity notwithstanding, in common, these stromal cells are individually surrounded by fibronectin, anchored by α5/β1 integrin, and often express COX-2 protein.

The initiation of mCMV/SMG fibroblast interaction is a function of viral binding to the cell surface and viral entry following fusion of its envelope to the cell membrane [59]. Subsequent to viral entry, virion components, including the viral genome, are rapidly transported to the host cell nucleus for viral transcription and replication. mCMV expresses ~200 viral products in a temporal, cascade-like manner: immediate early (IE), early, and late.

To achieve its goals, the virus co-opts the host cell genome and proteome. In the process, CMV infection of fibroblasts inhibits cell cycle progression [60,61], as well as cell death [27,29-32]. Thus, the infected host cell becomes a homeostatic "factory" for viral replication and paracrine signaling to uninfected SMG fibroblast and epithelial cells nearby or distant (Fig. 12). Replication of active mCMV is essential to the initiation and maintenance of embryonic SMG pathogenesis (Figs. 8, 9). The primary response to aberrant paracrine (and perhaps juxtacrine) signaling is the transcription factor-mediated up or down regulation of downstream genes. Our initial investigation has identified several important transcripts in this regard (Table 1).

Studies of the global and local properties of transcription-regulating networks reveal that the number of target genes regulated by each transcription factor follows a power-law distribution, i.e. a small number of hub nodes are connected to a very large number of other nodes (see review [62]). One such hub critical to regulation of embryonic SMG cell proliferation and apoptosis is NFκB [44]. NFκB signaling occurs via two independent pathways, the canonical NFκB1(p50)/RelA and the noncanonical NFκB2(p52)/RelB.

It is well established that mCMV and hCMV induces canonical NFκB during infection in fibroblasts, as well as other cell types, and this activation facilitates viral replication in some but not all settings [56,63-66]. It appears that viral IE1 activates NFκB by inducing nuclear localization, rather than transcript upregulation [55,56]. Reciprocally, there are NFκB recognition sites in the promoter and enhancer regions of *ie *genes [67]. Here we report that viral IE1 is expressed in the stromal cells of mCMV infected embryonic SMGs (Fig. 4), and that there is a coincident significant upregulation of noncanonical RelB and NFκB2 (p100/p52) transcript (Table 1). Recently, it has been shown that IE1 also induces the transcription of *Relb*, and that induction of the *Relb *promoter is mediated by JNK activation of AP-1 [51,52]. Thus, it is reasonable to expect that both pathways are activated and contributing to the pathogenesis of embryonic SMGs (Fig. 12).

Even if so, it is not without its complexity. Recently, Benedict et al. [56] have shown that for CMV replication in cultured fibroblasts, activation of the canonical NFκB1(p50)/RelA pathway and binding of p50/RelA to the major *ie *gene promoter is dispensable. Indeed, RelA may even suppress the potentiation of mCMV replication. The studies reported here in whole SMG explants support these findings (Fig. 10). Thus, while the canonical NFκB pathway may be an early participant in host cell mCMV replication and pathogenesis, paradoxically, it also buffers both.

IL-6, an NFκB target, is a multifunctional cytokine that mediates cell proliferation through the JAK-SHP2 pathway and cell terminal differentiation and survival through the JAK-STAT pathway [68,69]. IL-6 and its cognate receptors (IL-6R, gp130) are normally expressed only in SMG epithelia from the *Canalicular *Stage to the *Late Terminal Bud *Stage, as are STAT3 and bcl2; IL-6 signaling is an important factor in SMG developmental homeostasis [34]. mCMV replication in embryonic SMGs results in an enhanced expression of IL-6 protein in poorly proliferating ductal epithelia (Fig. 5), as well as a significant upregulation of *Il6 *(23-fold) and *Stat3 *(~2-fold) transcript and downregulation of *Erk1 *transcript (Table 1).

Taken together, these findings are dispositive of the histopathology (Figs. 1, 5): early diminished cell division and exaggerated maturation of ductal epithelia, and later epithelial invasion of the ductal space and stroma. The former is entirely consistent with the well-documented observation that STAT3 levels are key to the cellular choice between proliferation and maturation, low levels favoring proliferation and higher levels of maturation (see review [70]). As to the latter, among STAT family members, EGF-R-dependent and EGF-R-independent (i.e. IL-6) constitutive activation of STAT3 is the most frequently associated with deregulated (anti-apoptotic) cell growth and neoplasia [71]. Finally, it should be noted that the upregulation of STAT3 is not necessarily related to the upregulation of IL-6. Prior IL-6 gain of function studies reveal a dramatic increase in epithelial branching consequent to a 3-fold increase in epithelial cell proliferation, i.e. induction of the mitogenic JAK-SHP2 pathway, not the JAK-STAT3 maturation pathway [34].

COX-2 is another NFκB target. Here we report that, with mCMV infection, there is a highly significant, 15-fold, increase in *Cox2 *transcript (Table 1), as well as a dramatic appearance of COX-2 protein first in infected fibroblasts and later in metaplastic stromal cells (Fig. 7). COX-2 converts arachidonic acid to the intermediate PGH2, which is then converted to PGE2 by PGE synthase; PGE2 release from cells and binding to EP receptors, results in a broad activation of cAMP with downstream effects similar to protein kinase inducers (see review [72]). In the mCMV associated pathogenesis of SMG dysplasia, NFκB-mediated upregulation of COX-2 is most probably both mCMV-induced and pathology-induced (Fig. 12).

Investigating the relationship between CMV infection, induction of COX-2, synthesis of PGE2 and viral replication, Zhu et al. [41] found that after exposure of fibroblast cells to CMV, the synthesis and release of PGE2 becomes maximally elevated within the first 24 hrs, well before release of progeny virus has begun. This is accompanied by a dramatic increase in COX-2 protein in infected fibroblasts. COX-2 inhibition by specific inhibitors results in a downregulation of many viral transcripts and proteins, including the IE transcriptional activator; consequently, viral DNA synthesis and replication is substantially blocked. Thus, elevated levels of COX-2 and PGE2 are required for efficient replication of CMV in fibroblasts. Our present results would suggest the same is likely in SMG fibroblasts (Fig. 7C).

Additionally, with progressive mCMV-induced SMG pathogenesis, one finds proliferating metaplastic stromal cells individually anchored to fibronectin (FN) by α5/β1 integrin; many of these cells express COX-2 protein and display nuclear localization of β-catenin (Figs. 1, 7). Regardless of origin, the metaplastic stromal cells form α5/β1 integrin complexes and deposit the cognate ligand, FN. This large, extracellular matrix protein is assembled in elastin fibrils and subjected to contractile forces [73]. α5/β1 integrins serve as the interface between extracellular tensile cues and biochemical signals in the cytosol [74], including cell proliferation and cell survival [75,76]. Our findings suggest the likely function in metaplastic stromal cells of a recently described FN signaling cascade: FN → α5/β1 → MAPK → NFκB → COX-2 ([38]; Fig. 12). COX-2 mediated PGE2 signaling would directly activate β-catenin nuclear translocation and the expression of survival and growth-promoting genes [39], and perhaps the transactivation of tyrosine kinase receptors as well [77].

Finally, there is histologic and immunohistologic evidence (Figs. 2, 6, 7) that suggests mCMV-induced epithelial emigration and metaplasia – a kind of epitheliomesenchymal transition (EMT): dissociation towards single, disseminating polygonal cells with strong nuclear accumulation of β-catenin. This is not unlike some premalignant and malignant lesions (e.g. [78]). To be sure, *ex vivo*, whole organ verification of EMT is difficult to assay because of the transient and reversible nature of the process per se, and the lack of definitive markers that distinguish "neoplastic" cells undergoing EMT from neighboring stromal fibroblasts [79]. Nevertheless, the expression of epithelial-specific proteins (cytokeratin, E-cadherin, p120 and mucin) in a subpopulation of stromal cells would appear indicant, and could reasonably explain why CMV-induced stromal changes closely phenocopy oncocytic metaplasia.

The relationship between CMV and cancer has been confusing, contradictory, and controversial. Thirty years ago, Geder et al [80] reported the oncogenic transformation of human embryonic lung cells by hCMV. The tumors were composed of small, poorly differentiated, polygonal cells with large nuclei and scanty cytoplasm embedded in abundant matrix. In the ensuing years, the debate has been well-joined (see e.g. [81-85]). mCMV-induced, oncocytic-like, metaplasia and atypical ductal epithelial hyperplasia in embryonic SMGs suggest that the relationship between CMV and salivary gland tumors deserves a fresh look, particularly since salivary glands are a primary target for productive infection, subsequent latency, and reactivation.

## Conclusion

In summary, mCMV infection of embryonic mouse SMG explants results in dysplasia, metaplasia, and possibly, anaplasia. Initial investigation indicates that the molecular pathogenesis centers around the activation of canonical and, perhaps more importantly, noncanonical NFκB. Further, COX-2 and IL-6 are key downstream effectors of embryopathology. At the cellular level, there appears to be a consequential interplay between the transformed SMG cells and the surrounding extracellular matrix, resulting in the nuclear translocation of β-catenin. Much is obviously unknown. Nevertheless, a tentative framework has emerged (Fig. 12) within which additional studies may be planned and performed to clarify the spatiotemporal molecular pathogenesis.

## Methods

### Embryonic SMG culture system and mCMV infection

Female B10A/SnSg mice, obtained from Jackson Laboratories (Bar Harbor, ME), were maintained and mated as previously described [22]; plug day = day 0 of gestation. Timed-pregnant females were sacrificed on gestation day 15 (E15) and embryos were dissected in cold phosphate-buffered saline (PBS). All animal studies were conducted with the approval of the appropriate committees regulating animal research. An Animal Review Board and a Vivaria Advisory Committee review all applications to ensure ethical and humane treatment.

E15 SMG (mostly *Canalicular *Stage) primordia were cultured using a modified Trowell method as previously described [34,44,45]. The defined media consisted of BGJb (Invitrogen Corporation, Carlsbad, CA) supplemented with 0.5 mg ascorbic acid/ml and 50 units/ml penicillin/streptomycin (Invitrogen Corporation), pH 7.2; media was changed daily. Since notable differences in SMG branch number and size are usually seen among littermates, we employed a paired-design which compared right and left glands (treated and control) from each embryo.**mCMV infection**: on day 0, E15 SMGs were incubated with 10,000 to 200,000 plaque-forming units (PFU)/ml of *lacZ*-tagged mCMV RM427^+ ^[86] for 24 hrs and then cultured in virus-free BGJb defined media for an additional 2–11 (E15 + 3 to E15 + 12) days. Explants were collected and processed for whole mount morphology, routine histology, immunohistochemistry or multigene expression.

For whole mount morphological and size analyses, SMGs were photographed using a Wilde dissecting microscope at 25× and the area of each gland was determined using Image-Pro Version 4.0 (Media Cybernetics, Silver Spring, Maryland). The following groups were analyzed: 10,000 PFU: E15 + 3 (n = 7), E15 + 6 (n = 18); 50,000 PFU: E15 + 3 (n= 11), E15 + 6 (n = 12); 100,000 PFU: E15 + 3 (n = 17), E15 + 6 (n = 29), E15 + 12 (n = 5); 200,000 PFU: E15 + 3 (n = 6); E15 + 6 (n = 8). The significance of area differences between viral-infected and control SMGs was determined by Student t-test using a matched-pairs design.

For histological analysis, SMGs were fixed for 4 hrs in Carnoy's fixative at 4°C or overnight in 10% neutral buffered formalin at room temperature, embedded in paraffin, serially-sectioned at 8 μm and stained with hematoxylin and eosin as previously described [22,49]. For each experimental protocol, 3–20 SMGs/mCMV concentration/day were analyzed.

### mCMV analysis

To obtain a measure of mCMV infection, we assayed for β-galactosidase (*lacZ*) activity, viral titer/gland and localization of viral immediate early (IE1) proteins. **β-galactosidase (β-gal) staining**: Paired E15 + 3, E15 + 6 and E15 + 12 SMGs were fixed for 20 min at room temperature in 0.2% gluteraldehyde in PBS, washed 3 times in rinse solution (0.005% Nonidet P-40 and 0.01% sodium deoxycholate in PBS), stained for 4–6 hrs at room temperature in standard staining solution (5 mM potassium ferricyanide, 5 mM potassium ferrocyanide, 2 mM MgCl_2_, 0.4% X-gal in PBS), rinsed twice in PBS and microphotographed at 25×. β-gal whole mounts were then dehydrated through graded alcohols, embedded in paraffin, serially-sectioned at 8 μm and counterstained with eosin. **Viral titer**: E15 + 3 and E15 + 6 SMGs were collected, stored at -80°C in DMEM shipping media [87] and titered by plaque assay as previously described [87]. **IE1 distribution**: SMGs were fixed in Carnoy's fixative, serially-sectioned at 8 μm, and incubated overnight with anti-IEI as previously described [88]. Controls consisted of sections incubated with mouse IgG alone. For each experimental protocol, 3–10 SMGs/mCMV concentration/day were analyzed.

### Cell proliferation assay

Cell proliferation was determined by the localization of PCNA (proliferating cell nuclear antigen) as previously described [44]. Briefly, paired E15+ 3, E15 + 6 and E15 + 12 SMGs were fixed in Carnoy's fixative, serially-sectioned, incubated with anti-PCNA using the Zymed mouse PCNA kit (Invitrogen Corp.) and counterstained with hematoxylin. In this experiment, the cytoplasm appears blue and PCNA-positive nuclei appear dark brown. Three sections per slide and 3–5 SMGs per group were analyzed.

### Antibodies and immunostaining

Immunolocalization was conducted essentially as previously described [34,44,45]. The following monoclonal (Mab) and polyclonal (Pab) antibodies were used: Mab ATP-synthetase (Mitochondria marker) (#MAB3494, Chemicon International, Temecula, CA); Mab α5-integrin (# 103801, Biolegend, San Diego, CA); Mab β1-integrin (# 102201, BioLegend); Pab β-catenin (# AB19022, Chemicon International); Pab cytokeratin (# ab9377, Abcam Inc., Cambridge, MA); Pab COX-2 (# 160106, Cayman Chemical Company, Ann Arbor, MI); Mab E-cadherin (# 610181, BD Biosciences, San Jose, CA); Pab FN (# F3648, Sigma-Aldrich Corp., St. Louis, MO); Pab IL-6 (# sc-1265, Santa Cruz Biotechnology, Inc., Santa Cruz, California); Pab p120 (# sc-1101, Santa Cruz Biotechnology, Inc); Pab mucin [35]. For immunofluorescent analyses, Pab's were incubated with biotin-labeled rabbit IgG or anti-goat IgG (MP Biomedical, Aurora, OH) and then with Alexa-Fluor-labeled streptavidin (Invitrogen Corporation). Mab's were incubated with biotin-labeled anti-mouse IgG or anti-rat IgG (Jackson Laboratories, West grove, PA) and then with Alexa-Fluor-labeled streptavidin. Hamster antibodies (β-1 integrin) were incubated in FITC-labeled hamster IgG (Biolegend). Nuclei were counterstained with DAPI (Invitrogen Corporation). Immunohistochemistry was conducted essentially as previously described, using the Chemicon Tissue Staining Kit. Sections were viewed on a Zeiss Axioplan Microscope and photographed using 10×, 20× and 40× objectives. Confocal images were obtained using a Zeiss LSM-510 laser scanning confocal/multiphoton microscope (Carl Zeiss Inc. Thornwood NY) and the accompanying LSM version 3.2 image acquisition and analysis software. Cells were imaged using a plan-neofluor 1.3 numerical aperture, 40× objective lens. Alexa-Fluor- and FITC-labeled images were captured using a 488 nm Argon laser for excitation and a 505–530 nm band-pass filter to detect emission. DAPI images were captured using a 800 nm Mira titanium sapphire laser for excitation and a 390–465 nm band-pass filter to detect emission.

### CMV replication and pathology

Acyclovir, a synthetic purine nucleoside analgogue, is a highly selective agent for CMV with low toxicity to the host cell [53]. Acyclovir sodium (100 mg/20 ml) was purchased from American Pharmaceutical Partners, Inc (Schaumberg, Il). Since acyclovir has been shown at high doses to be teratogenic to rat embryos [89-91], we first determined the highest dose that is not teratogenic to E15 SMGs *in vitro*. Paired E15 SMGs were cultured in acyclovir (10 μg/ml, 20 μg/ml, 50 μg/ml or 100 μg/ml) or control medium for 3 (E15 + 3) or 6 days (E15 + 6) and acyclovir and control SMGs were compared by whole mount and histological analyses. Since doses ≥ 20 μg/ml acyclovir inhibited SMG branching and development, we employed 10 μg/ml acyclovir in all future experiments. Since no significant size differences were seen in SMGs infected with 50,000, 100,000 or 200,000 PFU mCMV, we infected SMGs with 50,000 PFU mCMV in this set of experiments. In the first experiment, we compared paired E15 SMGs infected with 50,000 PFU mCMV for 24 hrs and then cultured in control medium +/- 10 μg/ml acyclovir for a total of 6 days (CMV v. CMV + Acy) in culture; controls consisted of paired E15 SMGs cultured in control medium for 24 hrs and then in control medium +/- 10 μg/ml acyclovir for a total of 6 days (CONT v. Acy). E15 + 6 SMGs were collected and analyzed for whole mount morphology, mCMV infection (β-gal staining, titer determination), histopathology, and cell proliferation as described above. In the second experiment, E15 SMG explants were infected with mCMV for 72 hrs and cultured for an additional 3 (E15 + 6) or 9 (E15 + 12) days in the presence or absence of 10 μg/ml acyclovir (CMV v. CMV + Acy3). Controls consisted of E15 SMGs cultured in control medium (CONT) or control medium to which 10 μg/ml acyclovir was added after 72 hrs (Acy3). E15 + 6 and E15 + 12 SMGs were analyzed for whole mount morphology, mCMV infection (β-gal staining), histopathology, and cell proliferation. For each assay, 3–15 SMGs/group/day were analyzed.

### SN50 inhibition of canonical NFκB nuclear translocation

The cell permeable peptide SN50 (Biomol Research, Plymouth Meeting, PA) has been shown to inhibit canonical NFκB translocation into the nucleus. Previous studies in our laboratory have demonstrated that 100 μg/ml SN50 results in significant inhibition of SMG development [44]. In this set of experiments, we compared E15 + 6 SMGs infected with 50, 000 PFU mCMV and cultured in the presence or absence of 100 μg/ml SN50 for the entire culture period; concurrent control and SN50-treated E15 + 6 SMGs were also compared. As an additional control experiment, we cultured mCMV-infected SMGs in the presence or absence of SN50M, the control peptide; no differences were seen between E15 + 6 mCMV-infected and mCMV-infected + SN50M explants. The SMGs were analyzed for mCMV infection (β-gal staining) and histopathology; 3–10 explants/group were analyzed.

### RT-PCR

Paired E15 + 6 SMGs infected with 100,000 PFU CMV or cultured in BGJb control medium were pooled (10–12 SMGs/sample) and stored at -80°C in Trizol (Invitrogen Corporation). The glands were homogenized using lysing matrix D (FastRNA Pro Green kit, Q-Biogene, Morgan Irvine, CA) and RNA was extracted using the Trizol protocol according to manufacturer. One microgram RNA was reverse transcribed into first strand cDNA using ReactionReady™ First Strand cDNA Synthesis Kit: C-01 for reverse transcription (Superarray Biosciences, Frederick, MD). The kit uses random primers (hexamers) and MMLV reverse transcriptase to reverse transcribe the entire population of RNA in an unbiased manner. Real time quantitative PCR was conducted with a BioRad iCycler^® ^using primers and templates mixed with the PA011 master mix (RT^2 ^Real-Time™ SYBR Green/Fluorescein PCR master mix, Superarray Bioscences). The primer sets used were prevalidated to give single amplicons and purchased from Superarray Biosciences: Bcl2 (#PPM02918A-24), Caspase3 (#PPM02922A-22), c-myc (#PPM02924A-24), Cdh1 (#PPM03652A-24), Ctnnb1 (#PPM03384A-24), Cox2 (#PPM03647A-24), cyclinD1 (#PPM02903A-24), cyclinD2 (#PPM02900A-24), Egfr (#PPM03714A-24), Fn1 (#PPM03786A-24), Il6 (#PPM03015A-24), Jnk1 (#PPM03234A-24), Itga5 (#PPMO3609A-24), Itgb1 (#PPM03668A-24), Lef1 (#PPM05441A-24), Mapk3 (#PPM03585A-24), Mdm2 (#PPM02929A-24), Nfkb1 (#PPM02930A-24), Nfkb2 (#PPM03204A-24), p53 (#PPM02931A-24), Rela (#PPM04224A-24), Relb (#PPMO3202A-24), Stat3 (PPM04643A-24), TGFb1 (# PPM02991A-24), Tgfb2 (#PPM02992A-24), Tgfb3 (#PPM02993A-24), Tnf (#PPM03113A-24). Primers were used at concentration of 0.4 microM. The cycling parameters were 95°C, 15 min; 40 cycles of (95°C, 15 sec; 55°C, 30–40 sec and 72°C, 30 sec). Specificity of the reactions was determined by subsequent melting curve analysis. RT-PCRs of RNA (not reverse transcribed) were used as negative controls. GAPDH was used to control for equal cDNA imputs and the levels of PCR product were expressed as a function of GAPDH. The relative fold changes of gene expression between the CMV-infected and control glands were calculated by the 2^-ΔΔCT ^method. For each gene of interest, we analyzed 3–5 independent samples. Significant departures from the hypothesis that there are no differences between CMV-infected and control glands were determined by Student t-test.

## Authors' contributions

MM conceived and designed this study, participated in analysis of histopathology, performed the statistical analysis, and drafted the manuscript. TJ participated in experimental design, was involved in and coordinated all experiments, and helped draft the manuscript. GA prepared the histological sections, performed some of the morphology and cell proliferation experiments, and generated figures. ESM provided mCMV and participated in the experimental design. JH conducted viral titer assays and IE1 immunohistochemistry. All authors read and approved the final manuscript.
